# Mesenchymal stromal cells in the treatment of pediatric hematopoietic cell transplantation-related complications (graft vs. host disease, hemorrhagic cystitis, graft failure and poor graft function): a single center experience

**DOI:** 10.3389/fped.2024.1375493

**Published:** 2024-05-09

**Authors:** Maria Pérez-Torres Lobato, Maria Isabel Benitez-Carabante, Laura Alonso, Silvia Torrents, Nerea Castillo Flores, Maria Luz Uria Oficialdegui, Melissa Panesso, Carla Alonso-Martínez, Maria Oliveras, Berta Renedo-Miró, Joaquim Vives, Cristina Diaz-de-Heredia

**Affiliations:** ^1^ Department of Paediatric Oncology and Haematology, Vall D'Hebron University Hospital, Barcelona, Spain; ^2^ Vall D'Hebron Research Institute (VHIR), Barcelona, Spain; ^3^ Banc de Sang I Teixits, Barcelona, Spain; ^4^ Department of Pharmacy, Vall D'Hebron University Hospital, Barcelona, Spain; ^5^ Department of Medicine, Faculty of Medicine, Autonomous University of Barcelona, Barcelona, Spain

**Keywords:** mesenchymal stromal cells, hematopoietic stem cell transplantation, graft vs. host disease, graft failure, poor graft function, hemorrhagic cystitis, children

## Abstract

**Objectives:**

To describe mesenchymal stromal cells (MSCs) in the treatment of hematopoietic stem cell transplantation (HSCT) complications and to assess its safety and efficacy.

**Methods:**

Single-center retrospective study (2016–2023). Patients under 20 years who received MSCs for the treatment of HSCT-related complications were included.

**Results:**

Thirty patients (53.7% boys), median age at transplant 11 years (range 2–19) were included. MSCs indications were: graft-vs.-host disease (GVHD) in 18 patients (60%), of them 13 had acute GVHD (43.3%) and 5 chronic GVHD (16.7%); Grade 3–4 hemorrhagic cystitis (HC) in 4 (13.3%); poor graft function (PGF) in 6 (20%), 5 of them receiving MSCs with a CD34 stem cell-boost coinfusion; graft failure (GF) in 2 (6.7%), to enhance engraftment after a subsequent HSCT. Infusion-related-adverse-events were not reported. Overall response (OR) was 83% (25/30); 44% of responders (11/25) showed complete response (CR). OR for GVHD, HC, PGF and GF was 83.3%, 100%, 66.7% and 100% respectively. Response rate was 40% (95% CI: 20–55) and 79% (95% CI: 57–89) at 15 and 30 days. With a median follow-up of 21 months (IQR11–52.5), overall survival (OS) was 86% (95% CI: 74–100) and 79% (95% CI: 65–95) at 6 and 12 months post-MSCs infusion.

**Conclusion:**

In our study, the most frequent indication of MSCs was refractory aGVHD (43.3%). Response rates were high (OR 83%) and safety profile was good.

## Introduction

Advanced Therapy Medicinal Products (ATMP) represent a new category of medicines with a wide therapeutic potential for treating different types of hereditary and acquired diseases. ATMP encompass gene therapy, somatic cell therapy (SCT), and tissue-engineered products (1, 2). SCT refers to autologous or allogeneic cellular material that has been manipulated to change their biological characteristics and then is transferred into a patient for medical purposes (3–5). One type of SCT are mesenchymal stromal cells (MSCs). MSCs are multipotent, non-hematopoietic stem cells able to differentiate into various cell types, such as adipocytes, osteocytes, chondrocytes, and cells present in other connective tissues (6, 7). MSCs, which are present in adult and fetal tissues, can be derived from numerous sources such as adult bone marrow (BM), umbilical cord blood (UCB) or adipose tissue (8). However, BM and UCB are the most common sources for clinical use (9, 10). MSCs exhibit plasticity, self-renewal, immunomodulation, and anti-inflammatory properties. For example, they are able to secrete cytokines and growth factors at sites of tissue injury and inflammation or induce immunosuppressive effects by direct cell-to-cell interaction and paracrine signalling (11, 12). Additionally, they play an essential role in the BM niche, as they have the ability to support hematopoietic stem cell survival and proliferation (6). These properties have prompted their clinical use in the setting of regenerative medicine and hematopoietic stem cell transplantation (HSCT) (7, 8, 10).

HSCT is an established curative treatment modality for patients with severe hematological and non-hematological diseases (13, 14). Given the biological properties of MSCs, preclinical and clinical studies showed promising results in the treatment of HSCT complications, such as GVHD (15–17), GF, poor graft function (PGF) (18–21) and hemorrhagic cystitis (HC) (22). However, available clinical trials are scarce (15, 17, 23), and the efficacy of MSCs in a transplantation setting is so far unclear.

## Methods

### Study aims

The aim of this study was to describe the clinical use of allogenic MSCs in the treatment of HSCTs complications (GVHD, HC, GF or PGF) in the real-world practice.

The secondary objectives were to determine:

- The safety profile of MSCs during and immediately after infusion [infusion-related adverse events (AE)].
- The efficacy of MSCs therapy in terms of response rate [overall response (OR), complete response (CR), partial response (PR), probability of response, no response (NR)] and overall survival (OS).

### Study design and inclusion criteria

Retrospective descriptive study between January 2016 and January 2023. Patients under 20 years of age who had received MSCs for the treatment of the above-mentioned HSCT-related complications were included. They all had at least 3 months of follow-up after MSCs infusions. All patients were treated in a single-center Pediatric Bone Marrow Transplant Unit. Neither patient received MSCs within a clinical trial, but off label. Given that the efficacy of MSCs in the transplantation setting is still unclear, they received MSCs when first line-therapies failed, which is the standard of care in our center.

For acute and chronic GVHD, MSCs were used in patients diagnosed with steroid-refractory (SR) GVHD after at least two previous lines of treatment. During MSCs treatment, previous immunosuppressive therapies were not discontinued, unless clinically indicated.

For patients with HC, MSCs therapy was indicated in case of grade 3 or 4 HC. As with GVHD, previous supportive therapies were not discontinued during MSCs treatment.

For patients diagnosed with PGF or GF, indication of MSC therapy was made based on the definitions below (24, 25).

### Data sources and ethics

Anonymized clinical data were collected from the clinical records at Vall d’Hebron Hospital (VHH). Patients and/or legal tutors gave written consent for the transplantation procedure and to record patient´s anonymized transplantation-related data for studies. Legal tutors and/or patients signed the inform consent to receive compassionate use MSCs therapy. Good clinical practice according to Helsinki Declaration were applied. The study was approved by the Ethics Committee of VHH.

### Mesenchymal stromal cells

Non-crossed matched allogenic MSCs were used in all cases. MSCs doses were manufactured in Banc de Sang i Teixits (BST) Barcelona, Spain. The dose was the same for all indications. However, there could be a little variation of the viable cells/kg dose (between 0.7 and 1.3 × 10^6^), which depended on the manufacturing and culture expansion process. In this study, the median dose that patients received was 1.1 viable cells/kg (range 0.8–1.3 × 10^6^ cells/kg).

Considering that we treated different HSCT-complications and a standardized dose and infusion schedule for MSC therapy has not yet been established the dosing and infusion schedule was based on previous publications (25–28). MSCs infusion scheme for each post-HSCT complication is summarized in Table 1.

**Table 1:** MSCs therapy indications and infusion scheme.

	Previous treatments	Number of patients *N (%)*	MSCs infusion scheme
aGVHD	GC, ECP, RXL, MMF, infliximab, sirolimus	13 (43.3)	4 infusions on days: +1, +4, +11, +18
cGVHD	GC, ECP, RXL	5 (16.7)	
Graf failure	1–3 previous HSCT	2 (6.7)	2 infusions on days: +1 (co-infusion with HSCT), +15
Poor graft function	Eltrombopag, CD34 boost	6 (20)	2 infusions on days: +1 (co-infusion with CD34 boost), +15
Hemorrhagic cystitis	Cydofovir, intravesical: hyaluroic acid, E-aminocaproic acid/urokinase	4 (13.3)	3–4 infusions on days: +1, +7, +14, (+18)
All	NA	30	NA

GC, glucocorticoids; ECP, extracorporeal photopheresis; RXL, ruxolitinib; MMF, mycophenolate; HSCT, hematopoietic stem cell transplant; aGVHD, acute graft versus host disease; cGVHD, chronic graft versus host disease; NA, not applicable.

MSCs source was either umbilical cord or bone marrow. MSC from different tissue sources complied with minimal identity criteria established by the International Society for Cell and Gene Therapy (29).

Wharton's Jelly (WJ)-MSCs were derived, expanded and characterized following Good Manufacturing Practice (GMP)-compliant procedures and appropriate donor informed consent as reported extensively elsewhere (30–32). Briefly, a fragment of umbilical cord tissue was cut longitudinally and the WJ was scrapped with a surgical scalpel, spread uniformly over the plastic surface of a T-flask with re-closable lid (TPP, Trasadingen, Switzerland), and incubated for 30 min at 37°C. After the incubation, 10 ml of Dulbecco's modified Eagle's medium (DMEM; Gibco, Carlsbad, CA, USA) containing 2 mmol/L glutamine was added and supplemented with 2 × 10^4^ UI/ml penicillin (Invitrogen, New York, NY, USA), 20 mg/ml streptomycin (Invitrogen), 120 µg/ml amphotericin B (Invitrogen), and 10%–20% human serum B (hSerB, Banc de Sang i Teixits, Barcelona, Spain). After 2–5 days, a washing step with saline solution was performed, and 10 ml of fresh medium was added. From this point, the culture medium was replaced every 3–4 days. Cells were further expanded *in vitro* by seeding cell culture flasks at (1–3) × 10^3^ cell/cm^2^. When the total number of cells reached at least 5 × 10^6^, they were frozen in cryovials producing the master cell bank (MCB). Further expansion was performed after thawing for the generation of either working cell bank (WCB) or drug product (DP) directly. Final product (FP) was defined as the cell suspension resulting from thawing a DP, washed and conditioned for administration in patients.

MSC identity was confirmed as CD45^–^CD105^+^, CD31^–^CD73^+^, CD90^+^, HLA-DR^−^. MSC immune potency was confirmed by their capacity to inhibit proliferation of stimulated lymphocytes in co-culture.

Bone Marrow (BM)-MSCs were obtained under GMP conditions using a bioprocess that included a derivation step of MSC from BM aspirates and *ex vivo* expansion in an approximately 21-days. BM samples were harvested from the posterior iliac crest of donors and nucleated cells (NC) were isolated using an automated Sepax device (Biosafe) and Ficoll- Paque reagent (GE Healthcare). Then NC were washed and plated at 2 × 10^5^ cells/cm^2^ onto cell culture vessels (CellSTACK) with DMEM supplemented with 10%. All cultures were maintained at 37°C and 5% CO_2_ in humidified incubators. The medium was changed every 3–4 days and at day 10, cells were trypsinized using 0.05% Trypsin/ethylenediaminetetraacetic acid (EDTA; Gibco, Life Technologies), when 70%–90% confluence was reached. Then cells were reseeded at 1 × 10^3^ cells/cm^2^. Finally on day 21, cells were harvested and washed with a saline solution (Plasmalyte; Baxter) and were frozen in cryovials.

During the process, intermediate products and finally doses were analyzed to verify compliance with the established criteria according to GMP regulations (33). All MSC products tested negative for bacteria and *Mycoplasma*, and endotoxin levels were always below 0.5 EU/ml.

### Definitions

Acute GVHD (aGVHD) was graded according to MAGIC criteria (34), which goes from 1 to 4, depending on the number of organs involved [skin, liver, lower gastrointestinal tract (GI) and upper GI] and its severity. Chronic GVHD (cGVHD) was graded according to the 2014 NIH criteria (35, 36). The overall severity of cGVHD was classified as mild, moderate, or severe based on organ-specific grading.

For HC diagnosis (22, 37), bacterial and fungal infections were ruled out. HC was categorized as grade 1: isolated microscopic hematuria; grade 2: macroscopic hematuria; grade 3; macroscopic hematuria with blood clots; grade 4: hemorrhage causing blood transfusion dependence, urethral obstruction, or renal function damage.

Primary graft failure was defined as the lack of achievement of an absolute neutrophil count (ANC) 0.5≥ × 10^9^/L by day +28 after peripheral blood/bone marrow allo-HSCT and by day +42 after umbilical cord blood transplantation (24, 25, 38).

PGF was defined as: cytopenia in at least two hematopoietic lines [ANC <1 × 10^9^/L, platelet count <30 × 10^9^/L, hemoglobin (Hb) <10 g/dl] lasting for more than two consecutive weeks in the presence of stable donor chimerism and hypocellular bone marrow. Other explanations (such as disease relapse, drugs, or infections) were ruled out (24, 25, 38).

### Response and survival

OR was defined as the proportion of patients who presented a PR or CR to MSC therapy. For GVHD CR was reached when the disease resolved completely and no other treatments after MSC infusion were needed. PR was defined as improvement in staging of at least one involved organ, grade or score (in case of cGVHD) and at least steady state on other symptoms (39). For GF, CR was achieved when ANC ≥0.5 × 10^9^/L was recovered. PGF CR was defined as ANC >1 × 10^9^/L, platelet count >30 × 10^9^/L, Hb >10 g/dl (20). Response in at least 1 hematopoietic cell lineage was considered as PR. For HC, CR was achieved when gross hematuria disappeared. If it persisted but improved in at least one grade, it was was considered PR (38). Overall response, overall survival and event-free survival were calculated from first MSC infusion.

### Safety assessment

Patients were monitored throughout the MSC infusion and 4 h after the procedure; during the infusion, non-invasive continuous cardiac and respiratory monitoring was performed, and vital signs were recorded hourly for up to 4 h after infusion. Premedication was not administered to any patient.

Since this was not a clinical trial, but an observational retrospective study, only AEs occurring during 4 h after MSCs infusion were considered, as this information was specifically documented in the patient clinical charts. However, patients’ medical charts were carefully reviewed in search of other possible AEs during the follow-up period.

### Statistical analyses

Statistical analyses were performed in SPSS (version 28.0). Significance was set at a *p* level of 0.05 (two-tailed). Qualitative results were expressed as absolute and relative frequencies and quantitative variables, as median and interquartile range (IQR) or range. To test if quantitative data was normally distributed, Shapiro-Wilk Test was used. Mann-Whitney *U*-test was applied to analyze if time from 1st MSCs infusion to OR was associated with better response (PR or CR). Kaplan Meier (KM) estimator was used to evaluate and graphically display OS. Cumulative incidence was used to estimate the probabilities of partial and complete response. Probabilities were reported as percent with a 95% confidence interval (CI).

## Results

### Patient characteristics

Thirty patients (53.7% boys, 46.3% girls), median age at transplant 11 years (range 2–19) treated at a single institution were included. One patient was included twice (patient 2 Tables 2, 3), as she received MSCs for 2 different indications at separated time-periods; first she received two MSC infusion due to GF, and 2 months later, 3 weekly MSCs infusions were administered to treat aGVHD.

**Table 2:** MSC therapy in patients with GVHD.

Patients	PRE-MSCs Infusion			MSCs Infusion			POST-MSCs Infusion				
	HSCT indication	GVHD (a/c) and grading (G.)	Previous lines of treatment	IST at MSCs infusion	Time *(days)* from diagnosis to 1st MSCs infusion	Number of infusions	Maximum response to MSCs	Days tomaximum response to MSCs	Loss of response and reactivation	Months of FU from 1st MSCs infusion	Clinical status at last follow up/time off- IST *(days)*
1	Secondary MDS	G.III (skin 3, LGI 2) aGVHD	4	GC, MMF	120	4	PR	28	Yes: severe cGVHD (skin, GI and lung)	6	Dead. Severe cGVHD (skin, GI and lung) /on IST
2	B-Thal	G.IV (skin 4, liver 2) aGVHD	4	GC, MMF, ECP	98	2	PR	25	Yes: severe cGVHD (skin, GI, lung and hepatic)	7	Dead. Severe cGVHD (skin, GI, lung and liver) /on IST
3	T-ALL	G.IV (LGI 4, liver 2) aGVHD	4	GC, ECP	140	4	PR	32	No	70	Alive, no GVHD/80
4	SAA	G.IV (skin 4, LGI 4) aGVHD	3	Sirolimus, ECP	85	3	CR	16	No	67	Alive, no GVHD/150
5	AML	G.IV (LGI 4, liver 2) aGVHD	4	GC, ECP, RXL	110	4	PR	27	No	62	Alive, no GVHD/95
6	T-ALL	G.III (skin 2, LGI 3) aGVHD	3	GC, ECP, sirolimus	80	4	CR	24	Yes: mild cGVHD (skin)	62	Alive with mild cGVHD (skin)/on IST
7	SDS	G.IV (skin 4, LGI 2) aGVHD	4	GC, ECP	100	4	PR	35	Yes: cutaneous G.III aGVHD	52	Alive, no GVHD/60
8	B-ALL	G.III (skin 3, LGI 2) aGVHD	4	GC, CsA, ECP, RXL	150	4	PR	30	No	52	Alive, no GVHD/70
9	x-ALD	G.IV (LGI 4) aGVHD	4	GC, CsA, RXL	110	4	NR	-	NA	51	Dead. G.IV aGVHD (skin 3, LGI 4) /on IST
10	B-ALL	G.IV (LGI 4) aGVHD	4	CsA, ECP, ruxolitinib	120	4	CR	21	Yes: cutaneous G.II-III aGVHD	26	Alive, no GVHD/35
11	B-ALL	G.IV (skin 4, LGI 4, liver 3) aGVHD	3	GC, ECP	60	4	NR	-	NA	17	Dead. G.IV aGVHD (skin 4, liver 2, LGI 3) /on IST
12	AML	G.II (skin 3) aGVHD	2	GC, CsA, ECP	50	4	PR	28	No	3	Alive with G.II aGVHD (skin)/on- IST
13	PID	G.II (skin 2, LGI 2) aGVHD	2	GC, CsA, ECP	45	4	CR	21	No	24	Alive, no GVHD/108
14	CGD	Moderate cGVHD (skin)	3	GC, CsA, ECP, RXL	74	4	PR	26	No	21	Alive with mild cGVHD (skin)/on IST
15	AML	Severe cGVHD (skin, joint and GI)	4	GC, ECP, RXL	70	4	PR	32	Yes: moderate cGVHD (skin)	21	Alive with moderate cGVHD (skin)/on IST
16	CGD	Severe cGVHD (skin)	3	GC, ECP, RXL	65	4	NR	-	No	19	Alive with moderate cGVHD (skin)/on IST
17	SAA	Moderate (skin, GI and liver) cGVHD	3	GC, CsA, ECP	60	4	PR	27	No	18	Alive with mild cGVHD (skin)/on IST
18	B-ALL	Moderate (skin and GI, liver) cGVHD	3	GC, MMF, ECP, RXL	75	4	PR	27	No	17	Dead. mild cGVHD (skin and GI) at the time of death/on IST

HSCT, hematopoietic stem cell transplant; aGVHD, acute graft versus host disease; cGVHD, chronic graft versus host disease; G, grading; IST, immunosuppressive therapy; FU, follow-up; MDS, myelodysplastic syndrome; SAA, acquired severe aplastic anemia; B-Thal, beta thalassemia major; AML, acute myeloid leukemia; ALL, acute lymphoblastic leukemia; SDS, Schwachman Diamond Syndrome; ALD, x-linked adrenoleukodystrophy; PID, primary immunodeficiency; GCD, chronic granulomatous disease; LGI, lower gastrointestinal tract; GC, glucocorticoids; ECP, extracorporeal photopheresis; RXL, ruxolitinib; CsA, cyclosporine; MMF, mophetil mycophenolate; CR, complete response; PR, partial response; NR, no response; NA, non-applicable.

**Table 3:** Evaluation of response to MSC therapy according to the indication of MSCs.

	Days from 1st MSCs infusion to OR *Median (IQR/range)*	OR *(n/N, %)*	CR *(n/N, %)*	PR *(n/N, %)*	NR *(n/N, %)*	Loss of response *(n/N, %)*
aGVHD	15 (range 7–23)	11/13 (84.6)	4/11 (36.4)	7/11 (63)	2/13 (15.4)	5/11 (45.5)
cGVHD	21 (range 12–30)	4/5 (80)	–	4/4 (100)	1/5 (20)	1/4 (25)
HC	9.5 (range 9–16)	4/4 (100)	2/4 (50)	2/4 (50)	–	–
GF	19.5 (range 12–27)	2/2 (100)	2/2 (100)	–	–	–
PGF	26 (range 13–34)	4/6 (66.7)	3/4 (75)	1/4 (25)	2/6 (33.3)	–
All	16 (IQR 9.75–22.25)	25/30 (83)	11/25 (44)	14/25 (66)	5/30 (17)	6/30 (20)

aGVHD, acute graft versus host disease; cGVHD, chronic graft versus host disease; HC, hemorrhagic cystitis; GF, graft failure; PGF, poor graft function; OR, overall response; CR, complete response; PR, partial response; NR, no response. NR, no response.

Patients received an HSCT due to a malignant disease (*n* = 16, 53.3%) or a non-malignant disease (*n* = 14, 46.6%). Most common malignant condition was acute lymphoblastic leukemia (*n* = 13, 81.2%) and most frequent non-malignant disorders were bone marrow failure (*n* = 4, 28.5%) and primary immunodeficiency (*n* = 4, 28.5%).

### MSCs indications, characteristics and dosage

MSCs therapy indications and infusion scheme are summarized in Table 1. MSCs therapy and patients characteristic for each specific indication is summarized in Supplementary Table S1 (HC), Supplementary Table S2 (GF and PGF) and Table 2 (GVHD). There were two patients with GVHD that did not strictly followed the infusion scheme for his/her specific MSCs indication (patients 2 and 4, Table 2); patient 4 received 3 doses instead of 4, as he showed CR shortly after the second MSC infusion. Patient 2 received 2 MSCs doses, as she has previously got 2 MSCs infusion due to graft failure.

MSCs source was umbilical cord (56.7%) or bone marrow (43.3%). The median number of infusions was 4 (IQR 2–4).

MSCs indications were: GVHD in 18 patients (60%), 13 of them had aGVHD and 5 cGVHD; Grade 3–4 HC in 4 patients (13.3%), all cases being due to BK virus; PGF in 6 patients (20%), of whom 5 were co-infused with a CD34 + stem cell boost; primary GF in 2 (6.7%), to enhance engraftment after a subsequent HSCT. All patients receiving a boost of CD34 cells were non-conditioned and the CD34 boost was administered on the same day of first MSC infusion. For both patients with GF, MSC first infusion was administered on the same day of the HSCT. The second MSC infusion was administered 2 weeks later (please see dosing schedule for patients with GF in Table 1).

Patient 1 (Supplementary Table S2) had previously undergone 3 allo-HSCT. Due to GF diagnosis, she received a 4th allo-HSCT from a different donor (unmanipulated haploidentical donor) and same stem cell source (peripheral blood). Conditioning consisted of fludarabine, cyclophosphamide, anti-thymocyte globulin and rituximab. Graft vs. host disease (GVHD) prophylaxis consisted of cyclosporine and methotrexate. Patient 2 (Supplementary Table S2) had undergone a previous allo-HSCT. After GF diagnosis, she received a second transplant from a different donor (mismatched unrelated donor, 9/10) and same source (peripheral blood). Conditioning consisted of fludarabine, cyclophosphamide and anti-thymocyte globulin. GVHD prophylaxis consisted of cyclosporine and mophetil mycophenolate (MMF).

Regarding previous lines of treatment, in case of GVHD, median lines were 4 (range 2–5). Of note, cyclosporine (CsA) was not considered a line of treatment, as it was part of GVHD prophylaxis during HSCT in all patients. MMF was neither considered a new line of treatment when referring to “previous lines of treatment” if patients had received it as GVHD prophylaxis (patients 2, 3, 8, 13 and 17). MSCs therapy for GVHD is summarized in Table 2.

Before the indication of MSCs therapy for PGF, one patient received a boost of donor CD34 + cells 50 days before first MSC infusion and another was treated with Eltrombopag but none of them responded. Eltrombopag was discontinued one month before MSCs infusion.

### Response analysis

Response assessment is summarized in Table 3.

OR was 83% (25/30): 44% of responders showed CR (11/25) and 66% had PR (14/25). OR for aGVHD, cGVHD, HC, PGF and GF was 84.6%, 80%, 100%, 66.7% and 100% respectively.

Median time from first MSCs infusion to any OR was 16 days (IQR 10–22). For aGVHD it was 15 days (range 7–23), for cGVHD 21 days (range 12–30), for HC 9.5 days (range 9–16), for PGF 26 days (range 13–34) and for GF, 20 days (range 12–27), respectively. In case of GVHD, median time from first MSCs infusion to any OR in patients with CR was 8 days (IQR 12–20). Median time to maximum response in patients with GVHD and CR was 25 days (range −24).

**Figure 1:**
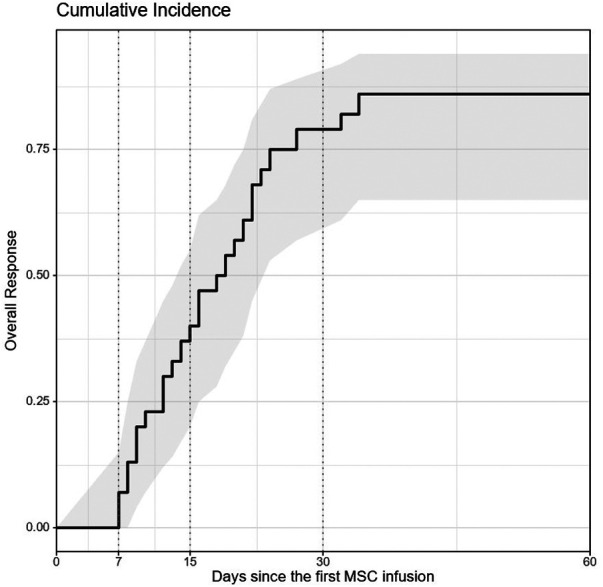
Kaplan-Meier plot probability of response. The x-axis represents days of follow up after the first MSC infusion, while the y-axis is the proportion of subjects who shows response after MSC therapy. Probability of response was 40% at 15 days (95% CI: 20–55), 79% at 30 days (95% CI: 57–89). Observe that, after 34 days, probability of response reaches a plateu and does not further increase.

The probability of response at 15 and 30 days was 40% (95% CI: 20–55) and 79% (95% CI: 57–89), respectively. After 34 days, probability of response reached a plateu [87.5%, 95% CI: 65–94)] and did not further increase (Figure 1). Five patients (17%) did not respond to MSC therapy. Loss of response (LR) was documented in 6 patients (20%) and median time from OR to LR was 2 months (IQR 2–5). All patients who showed LR had received MSCs due to GVHD.

When analyzing time from first MSCs infusion to any OR and type of response (PR/CR), statistical significance was observed, showing that patients with an earlier response had better response [12 days to OR (IQR 8–20.5) in cases of CR vs. 19 days to OR (IQR 15–23) in patients with PR; *p* = 0.038]. These differences were also observed when GVHD (acute and chronic) was analyzed separately [8 days (IQR 7–16) to CR vs. 20.5 days (IQR 16–23) to PR; *p* = 0.009].

### Safety

No patients experienced adverse events during or immediately after MSC infusion. No other AEs were retrospectively associated with MSCs therapy during the follow-up period.

### Survival analysis

**Figure 2:**
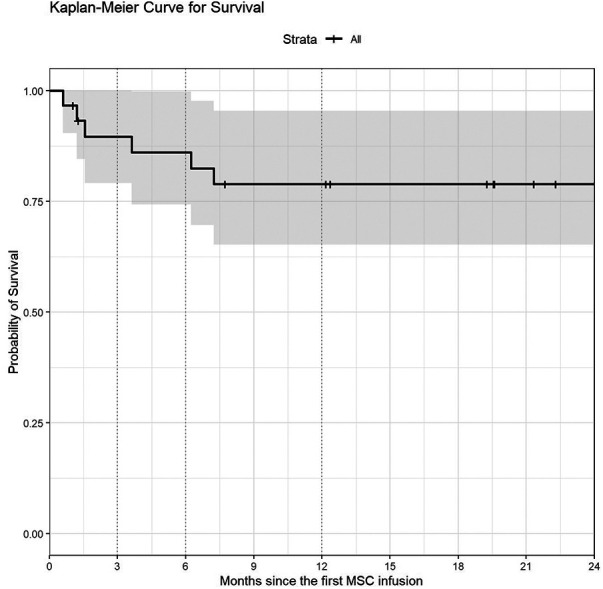
Kaplan-Meier plot of overall survival (OS) or probability of survival. The x-axis is months of follow up after the first MSC infusion until death occurs, while the y-axis is the proportion of subjects surviving. OS was 90% at 3 months (95% CI: 79–100), 86% at 6 months (95% CI: 74–100) and 79% at one year (95% CI: 65–95).

With a median follow-up of 21 months (IQR 11–52.5) from first MSCs infusion OS was 90% at 3 months (CI: 79–100), 86% at 6 months (CI:74–100) and 79% at twelve months (CI:65–95) post MSCs infusion (Figure 2). In case of GVHD (Supplementary Figure S1), OS was 82% (95% CI: 10–100), 77% (95% CI: 65–95) and 72% (95% CI: 59–889) at 6, 9 and 12 months respectively.

Overall survival for patients with NR was 53% (95% CI 21–100) at 6 and 12 months post MSCs infusion. In the PR group, OS were 80% (95% CI 60–100) and 60% (95% CI 36–100) at 6 and 12 months, respectively.

**Figure 3:**
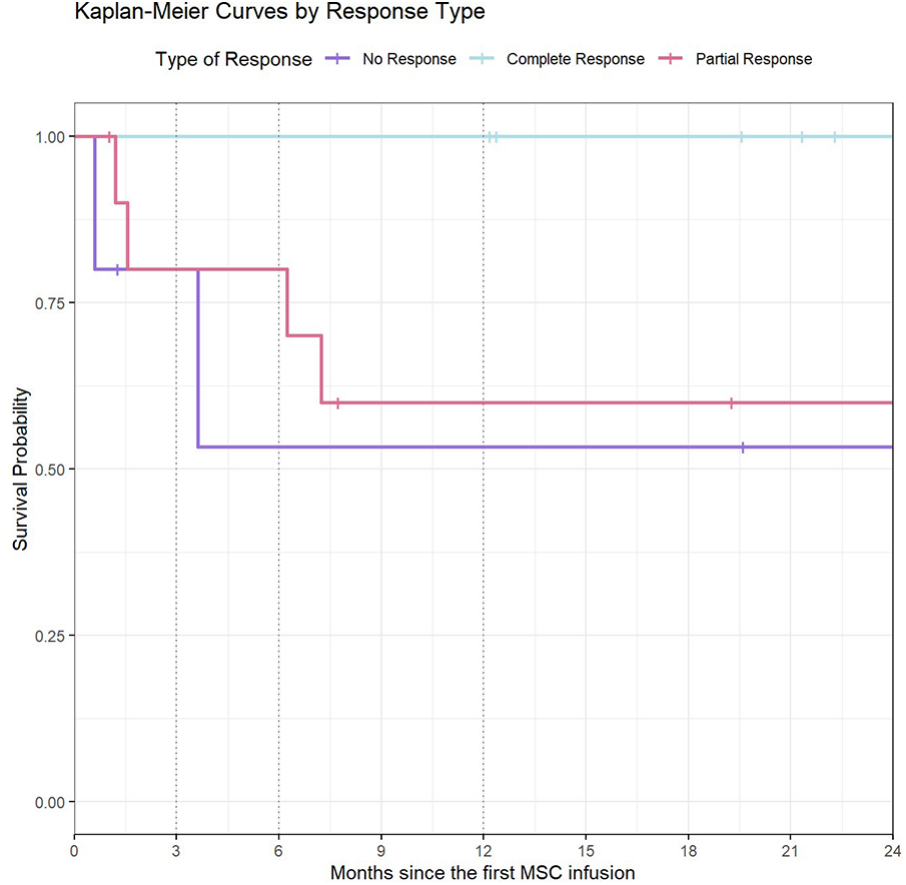
Kaplan-Meier curves by type of response. This graph represents the survival time (in months) from 1st MSC infusion, in relation to each type of treatment response. For patients with NR, OS was 53% (95% CI: 21–100) at 6 months of follow-up onwards. In the PR group, OS was 80% (95% CI: 60–100) at 6 months of follow-up and 60% (95% CI: 36–100) from 12 months onwards. Since no death were observed in the CR group, the survival probability was 100%.

Overall 7 patients (24%) died, but in none of them death was related to MSC therapy. No deaths were observed in the CR group (Figure 3). Causes of death are summarized in Table 4.

**Table 4:** Deaths and causes of death.

Patients	Diagnosis	MSCs indication	Maximun response to MSCs	Patient's clinical situation with respect to the indication that led to MSC therapy at last FU	Cause of death/months after last MSCs infusion
1	MDS	aGVHD	PR	Severe cGVHD	Respiratory failure: bronchiolitis obliterans/6
2^a^	B-Thal	aGVHD	PR	Severe cGVHD	Respiratory failure: alveolar hemorrhage/7
3	x-ALD	aGVHD	NR	aGVHD grade IV	Disease relapse/3
4	B-ALL	aGVHD	NR	aGVHD grade IV	Respiratory failure: alveolar hemorrhage/17
5	B-ALL	cGVHD	PR	Mild cGVHD	IFI/17
6	T-ALL	HC	PR	Grade 1 HC	Leukemia relapse/2
7	DKC	PGF	NR	PGF	Respiratory failure: complicated bacterial pneumonia, alveolar hemorrhage/54

MSD, myelodysplastic syndrome; B-Thal, Beta thalassemia major; ALL, acute lymphoblastic leukemia; ALD, x-linked adrenoleukodystrophy; DKC, Dyskeratosis congenita; aGVHD, acute graft versus host disease; cGVHD, chronic graft versus host disease; HC, hemorrhagic cystitis; PGF, poor graft function; LGI, lower gastrointestinal tract; PR, partial response; NR, no response; FU, follow-up; IFI, invasive fungal infection.

^a^ Also referred as patient 2 in Table 2.

## Discussion

In the present study, we describe MSCs as a therapy for HSCT-related complications in a series of 30 pediatric patients. The most frequent indication was refractory aGVHD (43.3%, *n* = 13/30). In general, response rates were high (OR 83%) and, similar to other studies (17, 18, 22), safety profile was good.

Regarding GVHD, clinical studies of MSC therapy in patients with SR-aGVHD have demonstrated favorable clinical response rates with an acceptable safety profile (40–45). For example, Kurtzberg et al. (46) recently reported on 241 children with grade II–IV SR-aGVHD who were on additional second-line treatments. The 28-day OR rate was 65% with a 14% CR. The 100-day OR was not assessed. In another study (47), the same group analyzed 54 children with primary SR-aGVHD who were naive to other immunosuppressant therapies and were treated with MSC product (remestemcel-L). A 70.4% OR was achieved, and response was sustained through day 100, including an increase in CR from 29.6% at day 28 to 44.4% at day 100. In the present study, patients with aGVHD had comparable response rates, with 84% OR and 36.4% CR. Nonetheless, 20% of all patients (4 of them with aGVHD) presented loss of response. Of note, two patients who received MSCs due to aGVHD developed severe cGVHD and died of respiratory failure. Although they had previously shown PR to MSCs, they were certainly at risk of developing cGVHD, since both of them had prior severe aGVHD (grade III and IV) (48). As opposed to the 2nd study of Kurtzberg et al. (42), patient population in our study were highly pretreated and refractory to previous treatments, which might have worsened response.Some authors (42, 44, 49) have also showed that, when MSCs were applied in an earlier stage of SR-aGVHD, the CR rates were higher. For example, Le Blanc et al. (42), in their study of a group of SR grade II-IV patients, described 55% CR and 16% PR. For that reason, taking into account the side effects of second line immunosuppression for GVHD and the good safety profile of MSCs (even in severely affected children), earlier use of MSCs therapy could be considered for patients with acute SR-GVHD.

Compared to aGVHD, there is limited use of MSCs therapy in cGVHD, probably because, in many cases, the chronic fibrotic processes of cGVHD are irreversible (49). In our study, MSCs therapy was more effective in aGVHD than in cGVHD (84% OR with 36.4% CR vs. 80% OR with no CR). Similar to previous report (49–52), most patients showed response, although only PR were observed.

Another indication for MSC is HC (22, 53, 54). Although there is limited clinical experience and it is mainly used in adults, some pediatric studies have shown promising results; for example, Tong et al. (22) reported thirteen pediatric patients with severe BKV-HC (grade 3–4) who presented 100% OR with rapid symptoms improvement. These results are consistent with our series, where OR was 100% (50% CR) and no loss of response was observed.

MSCs have also been used to reverse graft failure and enhance engraftment both in children and adults (18–21, 55, 56). In a meta-analysis by Li et al. (18), studies comparing MSC co-transplantation in allo-HSCT with allo-HSCT alone where analyzed (19 clinical trials), showing that MSC co-infusion generally improved engraftment without increasing mortality or relapse. In another study by Servais et al., (20). MSC were administered as a single i.v. infusion at a dose of 1–2 million(s) cells/kg body weight in patients with PGF. Within 90 days post-MSC infusion, OR was 53% with 37% CR, observing a response rate increase to 67% OR and 53% CR within 180 days after MSC infusion. In our series, all patients with GF and MSC co-transplantation presented CR. Compared to Servais et al., most cases of PGF received a CD34 + cells co-infusion, observing response rates were higher (OR 66% with 75% CR) and more rapid [26 days from first MSC infusion (range 13–34)]. Certainly, the CD34 + boost or the subsequent HSCT have most probably improved response rates in our study. Therefore, more evidence is needed to confirm the real impact of MSCs on engraftment and graft function, alone (in case of PGF) or as a co-adjuvant therapy.

Regarding survival analysis, and according to literature, OS rates varies between 50 and 80%, depending on MSCs indication and time-point of analysis, among others (18, 20, 22, 46, 47). In our study, OS was 90% at 3 months (95% CI: 79–100) and 79% at one year (95% CI: 65–95). Notably, similar to Kurtzberg et al. (47), we observed survival was higher in responders compared with non-responders [none of the responders died while, in the non-responder's group, OS was 53% (95% CI: 21–100) at 6 months of follow-up onwards]. For that reason, especially in the case of aGVHD (the most frequent MSCs indication in our study), considering that early use of MSCs seems to improve response and consequently, survival, with no identified safety concerns (even in gravely ill children such as our cohort), we believe that, despite possibly being a promising therapeutic tool, there is certainly room for improvement in the use of MSCs.

Regarding the causes of death (Table 4), three patients died of respiratory failure and alveolar hemorrhage. We believe these deaths were not related to MSCs therapy since MSCs infusions occurred more than 6 months before either death (7, 17 and 54 months in patients 2, 7 and 4 respectively). Additionally, all these patients had previous risk factors of pulmonary bleeding. For example, the patient with dyskeratosis congenital (patient 7, Table 4) maintained platelet count between 30 and 50.000 and developed empyema and pulmonary hemorrhage after chest tube drainage, which prompted death. The other two patients (one with beta-thalassemia and the other with B-acute lymphoblastic leukemia) developed severe cytomegalovirus pneumonitis and diffuse alveolar hemorrhage as a consequence, which led to death.

Limitations of our study stem in the small number of patients, the sample heterogeneity with variable follow-up periods, the retrospective design of the study, the lack of control group against which to assess the efficacy and safety of MSCs and the fact that it was single-center. Additionally, the possibility that some patients might have responded to other concomitant therapies rather than MSCs cannot be excluded. There are some confounding factors when comparing results between studies that we should also acknowledge, such as variability in MSCs donor types, MSCs dose per infusion, and number of infusions per patient—even within the same trial in some instances.

In conclusion, our study supports that MSCs seem to be a safe and effective therapy in the treatment of pediatric patients with HSCT-related complications. Further adequately powered prospective studies and clinical trials are required to confirm efficacy and establish the place and infusion schedule of MSCs therapy in each specific HSCT-related complication.

## Data Availability

The raw data supporting the conclusions of this article will be made available by the authors, without undue reservation.

## Supplementary material

Supplementary Figure S1 Kaplan-Meier curves in GVHD. This graph represents the probability of survival of patients with GVHD from 1st MSC infusion (purple curve), and the probability of survival of all the patients treated with MSCs in this study (black curve). For patients with GVHD, OS was 82% (95%CI: 10–100), 77% (95%CI: 65–95) and 72% (95%CI: 59-889) at 6, 9-and 12-months post MSCs infusion. For all the patients OS was a little higher; 90% at 3 months (95%CI: 79–100), 86% at 6 months (95%CI: 74–100) and 79% at twelve months (CI:65-95) post MSCs infusion.
