# Supplementary material for: Mesenchymal stromal cells in the treatment of pediatric hematopoietic cell transplantation-related complications (graft vs. host disease, hemorrhagic cystitis, graft failure and poor graft function): a single center experience
Source: Front Pediatr. 2024 May 9;12:1375493. doi: 10.3389/fped.2024.1375493 (PMC11112085; doi:10.3389/fped.2024.1375493)
Supplement: Supplementary file 1 [file Table1.docx]

**Supplemental table 1. MSC therapy in patients with HC**

|  | **PRE-MSCs INFUSION** | | **POST-MSCs INFUSION** | | | | |
| --- | --- | --- | --- | --- | --- | --- | --- |
| Patients | Previous treatments | uBK virus  *(N copies ×10^8^/mL)* | Days to gross hematuria resolution (days) | uBKvirus  *(N copies ×10^8^/mL)* | Maximal response to MSCs | Months of follow-up from MSCs infusion | Clinical status at last follow-up |
| 1 | Cydofovir, intravesical hyaluronic acid, E-aminocaproic acid/urokinase | 5.86 | 27 | 4.32 | PR | 3 | Death (leukemia relapse) |
| 2 | Cydofovir, intravesical hyaluronic acid, urokinase, continuous bladder irrigation | 4.48 | 23 | 3.36 | PR | 66 | No hematuria, normal renal function |
| 3 | Cydofovir, intravesical hyaluronic acid, continuous bladder irrigation | 3.32 | 12 | NA | CR | 80 | No hematuria, normal renal function |
| 4 | Cydofovir, intravesical hyaluronic acid, continuous bladder irrigation | 5.92 | 10 | 2.67 | CR | 24 | No hematuria, normal renal function |

uBK: urinary BK, NA: not available, PR: partial response, CR: complete response
