# Supplementary material for: Mesenchymal stromal cells in the treatment of pediatric hematopoietic cell transplantation-related complications (graft vs. host disease, hemorrhagic cystitis, graft failure and poor graft function): a single center experience
Source: Front Pediatr. 2024 May 9;12:1375493. doi: 10.3389/fped.2024.1375493 (PMC11112085; doi:10.3389/fped.2024.1375493)
Supplement: Supplementary file 2 [file Table2.docx]

**Supplemental table 2. MSC therapy in patients with GF and PGF**

|  |  | | **POST-MSCs INFUSION** | | | | |  |  |
| --- | --- | --- | --- | --- | --- | --- | --- | --- | --- |
| Patients | Diagnosis | Number of hematopoietic lineages affected | | Previous treatments | Blood product transfusions per week | Maximal response to MSCs | Months of follow-up from MSCs infusion | | Clinical status at last follow-up |
| 1 | PGF | 2 | | - | 3-4 | CR | 8 | | Transfusion indepence |
| 2 | PGF | 2 | | - | 2-3 | CR | 12 | | Transfusion indepence |
| 3 | PGF | 2 | | Eltrombopag | 1-2 | CR | 23 | | Transfusion indepence |
| 4 | PGF | 2 | | CD34 boost | 2-3 | PR | 19 | | One platelet transfusion every three weeks |
| 5 | PGF | 3 | | - | 3-4 | NR | 54 | | Death (respiratory failure) |
| 6 | PGF | 2 | | - | 3 | NR | 3 | | Received subsequent HSCT |
| 1 | Primary GF | 3 | | 3 allo-HSCT:  -1^st^ MSD, BM  -2^nd^ MSD, PB  -3^rd:^ MSD, PB | 4 | CR | 8 | | Death (respiratory failure) |
| 2 | Primary GF | 3 | | 1 allo-HSCT  MSD (PB) | 3-4 | CR | 43 | | Transfusion indepence |

PGF: poor graft function, GF: graft failure, HSCT: hematopoietic stem cell transplantation, MSD: matched sibling donor, PB: peripheral blood, BM: bone marrow, , CR: complete response, PR: partial response.
