# Supplementary figures and images for: Mesenchymal stromal cells in the treatment of pediatric hematopoietic cell transplantation-related complications (graft vs. host disease, hemorrhagic cystitis, graft failure and poor graft function): a single center experience
Source: Front Pediatr. 2024 May 9;12:1375493. doi: 10.3389/fped.2024.1375493 (PMC11112085; doi:10.3389/fped.2024.1375493)

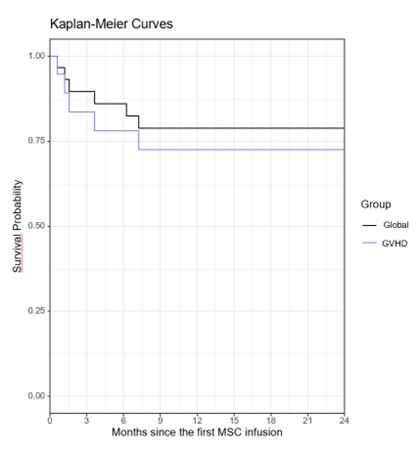

Supplement: Supplementary Figure S1 — Kaplan-Meier curves in GVHD. This graph represents the probability of survival of patients with GVHD from 1st MSC infusion (purple curve), and the probability of survival of all the patients treated with MSCs in this study (black curve). For patients with GVHD, OS was 82% (95%CI: 10–100), 77% (95%CI: 65–95) and 72% (95%CI: 59-889) at 6, 9-and 12-months post MSCs infusion. For all the patients OS was a little higher; 90% at 3 months (95%CI: 79–100), 86% at 6 months (95%CI: 74–100) and 79% at twelve months (CI:65-95) post MSCs infusion. [file Image1.jpg]
